# Landscape of Physical Activity and Quality of Life Research in Breast Cancer Survivors: Topic Modeling Analysis

**DOI:** 10.3390/jcm14165615

**Published:** 2025-08-08

**Authors:** Suryeon Ryu, Ki-Yong An, Min Song, Zan Gao

**Affiliations:** 1 Center for Children’s Healthy Lifestyles & Nutrition, Children’s Mercy Hospital, Kansas City, MO 64108, USA; sryu@cmh.edu; 2 Faculty of Kinesiology, Sport, and Recreation, University of Alberta, Edmonton, AB T6G 2H9, Canada; kiyong1@ualberta.ca; 3 Department of Library and Information Science, Yonsei University, 50 Yonsei-ro, Seodaemun-gu, Seoul 03722, Republic of Korea; min.song@yonsei.ac.kr; 4 Department of Kinesiology, Recreation, and Sport Studies, The University of Tennessee, Knoxville, TN 37996, USA

**Keywords:** cancer side effects, healthy lifestyles, functional assessment, psychosocial health, research trends, social support

## Abstract

**Background/Objectives:** Physical activity (PA) is widely recognized as a beneficial approach to improving the health-related quality of life (HRQoL) of breast cancer survivors. This study explored key research topics and emerging trends in studies related to PA and HRQoL among breast cancer survivors. **Methods:** Titles and abstracts of 3847 English-language research articles (2000–2024) were retrieved from PubMed, EMBASE, Web of Science, and Scopus using keywords related to ‘breast cancer’, ‘PA/exercise’, and ‘HRQoL’. A text-mining algorithm based on the Dirichlet-multinomial regression approach in Python was applied to identify the top 10 research topics and their trends over time. **Results:** In total, 10 key topics emerged: (1) Quality of Life and Well-being, (2) Cancer Treatment and Health-Related Fitness, (3) Supportive Care and Psychosocial Factors, (4) Survivorship, Palliative Care, and Integrative Medicine, (5) Physical Activity and Sedentary Behaviors, (6) Upper Limb-Related Side Effects, (7) Cancer-Related Fatigue and Symptoms, (8) Epidemiological and Clinical Factors, (9) Side Effects of Cancer Treatment, and (10) Weight Management. Among these, Topics 1, 2, 3, 8, and 9 followed upward trajectories, while others showed relatively stable trends. **Conclusions:** Findings highlight that PA research on breast cancer survivors’ HRQoL spans all stages of survivorship and considers both clinical outcomes and psychosocial and emotional well-being. Understanding how PA and HRQoL have been represented in research helps clarify which survivor needs have received attention and which remain underexplored. These thematic patterns underscore growing acknowledgement of survivors’ lived experiences and offer a roadmap for addressing future research and care gaps.

## 1. Introduction

Cancer remains a pressing global health issue and continues to be the second leading cause of death [1]. Among the various types of cancer, breast cancer notably ranks as one of the most widespread forms among the female population [2]. As projected by the American Cancer Society for the year 2025, approximately 316,950 new cases of breast cancer are anticipated to be diagnosed, with an estimated 42,170 deaths attributed to the disease in the Unites States [2]. Although breast cancer remains the primary cause of mortality among women, advancements in early detection and enhanced treatment options have led to improved survival rates. The five-year survival rate for breast cancer diagnosed at an early (localized) stage is 99%. With the increasing number of individuals living with cancer or in remission, there has been a growing focus in recent years on improving their health-related quality of life (HRQoL).

HRQoL is a multi-dimensional concept, encompassing physical, mental, and social well-being [3]. For cancer survivors, the process of readjusting to their new normal lives can be disconcerting from the time of diagnosis through the completion of major treatments [4]. These challenges often include a decline in physical capabilities, heightened mental health challenges such as depression and anxiety, and persistent worries about recurrence [4]. All of these health concerns are closely connected with HRQoL. Adopting an active lifestyle during survivorship is widely recognized as a key strategy for improving treatment outcomes, reducing the likelihood of cancer recurrence, and enhancing HRQoL [5]. Therefore, substantial efforts have been devoted to developing physical activity (PA)-based interventions to support breast cancer survivors (BCS). For example, Gao et al. found that engaging in light-intensity PA such as Tai Chi can effectively enhance mental well-being among BCS [6].

Despite the clear benefits of PA for BCS and growing attention to HRQoL in survivorship, relatively little work has synthesized the broader research landscape to identify emerging topics and trends. Understanding where scholarly efforts are directed and how they evolve can help researchers pinpoint underexplored issues, novel methodologies, and new theoretical frameworks, all of which guide the field toward the most pressing and impactful questions. Although traditional reviews are valuable, they are labor-intensive, prone to becoming outdated as new research emerges, and may overlook latent themes. Additionally, manual screening introduces the risk of selection bias.

Text-mining methods (e.g., Dirichlet-multinomial regression [DMR]) offer a powerful means to address this gap by uncovering hidden topics and temporal patterns in large volumes of textual data [7,8]. DMR not only identifies latent themes by incorporating metadata (e.g., publication year) as covariates, but also models how these themes evolve over time [8]. Its ability to detect nuanced patterns that might go unnoticed in traditional reviews or meta-analyses is particularly valuable in this rapidly changing field. By revealing how research has shifted, decision-makers and researchers alike can stay informed of the latest developments, pinpoint unexplored areas, and direct resources toward interventions that most effectively bolster HRQoL among BCS. To the best of our knowledge, no systematic investigation of research trends in HRQoL and PA among BCS has been conducted. This study is the first of its kind in the proposed research field. Thus, we employed DMR to achieve two primary aims: (1) to identify the primary research topics related to BCS, PA, and HRQoL and (2) to examine how these research trends have evolved since 2000.

## 2. Materials and Methods

### 2.1. Process

The research team finalized the search terms, and the first author retrieved bibliographic details (i.e., year of publication, title, and abstract) from four databases (i.e., PubMed, EMBASE, Web of Science, and Scopus) on 7 February 2025. The data (n = 4479) were then imported into the Rayyan platform to remove duplicates, and articles without abstract information were excluded. After verifying that each record contained complete bibliographic data, the first author performed text-mining analyses on a tab-delimited file containing the year, title, and abstract for each document (n = 4292). To focus on original research, review papers (n = 445) were removed, resulting in a final dataset of 3847 articles. Additionally, words that appeared excessively frequently (i.e., ‘very common’ words offering minimal distinction among articles) or provided limited meaningful value were designated as ‘stopwords’ to prevent skewing the data. Closely related terms were also merged to reduce repetitive noise. The topic labels were finalized through a systematic, collaborative process within the research team, including experts in movement behavior science, cancer survivorship, and exercise oncology. Initially, each team member independently reviewed the DMR-derived keywords and proposed preliminary labels. These were then discussed in a series of consensus meetings, where interpretations were compared, discrepancies addresses, and terminology refined. This iterative process was repeated until full agreement was reached on each label. Finally, the evolving research landscape was visualized to highlight recent research trends.

### 2.2. Data Collection

The comprehensive literature search combined three main concepts: (1) breast cancer, (2) PA/exercise, and (3) HRQoL. The search was applied to the titles, abstracts, and keywords of articles in English-language journals published between 1 January 2000 and 31 December 2024. After removing duplicates, articles lacking abstracts, and review papers (including scoping reviews, systematic reviews, and meta-analyses), a total of 3847 documents were identified and included in the subsequent text-mining analyses. The structure of search queries was the same across the four databases, and an example of the detailed search queries used in PubMed is presented in Supplementary Materials (Table S1).

### 2.3. Data Processing

**Figure 1:**
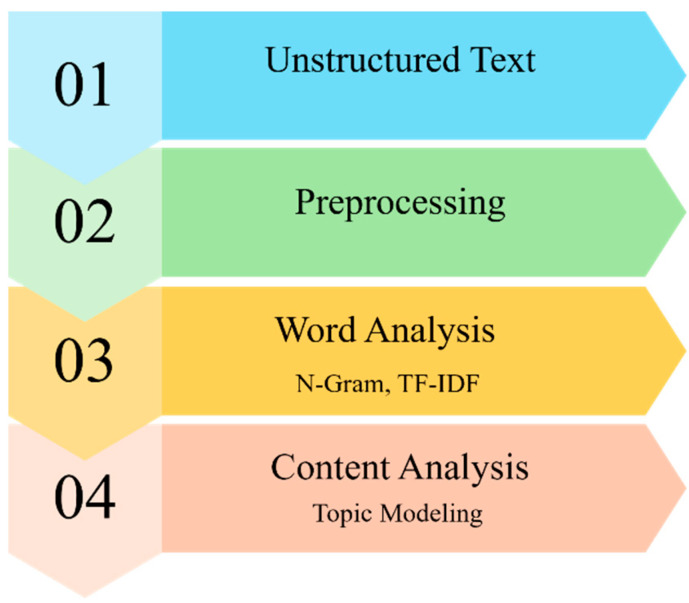
Data Process. Note: TF-IDF: Term Frequency–Inverse Document Frequency.

The overview of our data processing pipeline is illustrated in Figure 1. We used Python-based text-mining algorithms in Jupyter Notebook 7.2.2 (Python 3.12), passing the data through multiple text-cleaning and preprocessing steps (e.g., tokenization, lemmatization). During these steps, we removed stopwords and replaced certain multi-word expressions with single tokens (e.g., ‘physical function’ to ‘physical_function’) to reduce noise and prevent partial merges of key phrases. We also applied bigrams, re-segmenting the text into two-word n-grams. The final list of tokens was then used for both term frequency–inverse document frequency (TF-IDF) analysis and topic modeling. Before conducting the topic-model analysis, we tested a range of candidate topic counts and evaluated each using two standard fit metrics (i.e., perplexity and coherence). We then applied topic modeling algorithms (i.e., DMR via tomotopy 0.13) to generate thematic groupings by learning latent topics from the corpus. The DMR model produces two main outputs: (1) topic–word distributions, showing which words characterize each topic, and (2) learned regression coefficients (lambdas) for each topic–metadata (year) pair, indicating how strongly a given year correlates with a given topic. Because raw lambda coefficients can be negative or positive, we shifted them into positive territory for easier trend interpretation. All visualizations were generated in Python.

More specifically, we used DMR with document year as metadata, allowing us to explore latent research topics based on the model’s per-topic word distributions. We trained an unsupervised DMR model to discover 10 major themes, then examined how each theme varied over time using topic-year lambdas. We refined the DMR results iteratively by adding relevant stopwords (e.g., frequently occurring but contextually uninformative terms such as ‘breast cancer’, ‘health-related quality of life’, ‘quality of life’, ‘physical activity’, and ‘exercise’), guided also by TF-IDF outputs to identify low-discriminative terms for removal. Our analysis of research trends centered on how topics evolved over time, highlighting shifts in attention and thematic focus in the field. A topic’s trend line was determined by the transformed lambda, which indicated its association strength with each year. An upward trend in recent years suggests that the topic has become more dominant in later publications, whereas a downward trend suggests it was more prominent in earlier periods. For each topic, we also calculated the average change in each topic’s DMR coefficient using a simple linear regression of coefficient on publication year.

## 3. Results

### 3.1. Key Research Topics

Guided by acceptable perplexity (≈24,331) and coherence (≈0.45) scores, and reinforced by expert review, we selected 10 topics for the final DMR analysis. After applying the DMR model, 10 distinct topics related to BCS, PA/exercise, and HRQoL emerged. These topics are summarized in Table 1, with the full version provided in Supplementary Table S2. Because the DMR approach was unsupervised, we assigned labels to each theme based on the key terms within the documents. The resulting topics were as follows: (1) Quality of Life and Well-being, (2) Cancer Treatment and Health-Related Fitness, (3) Supportive Care and Psychosocial Factors, (4) Survivorship, Palliative Care, and Integrative Medicine, (5) Physical Activity and Sedentary Behaviors, (6) Upper Limb–related Side Effects, (7) Cancer-Related Fatigue and Symptoms, (8) Epidemiological and Clinical Factors, (9) Side Effects of Cancer Treatment, and (10) Weight Management. Supplementary Table S3 lists an example article corresponding to each topic.

#### 3.1.1. Topic 1: Quality of Life and Well-Being

This topic includes keywords associated with several validated questionnaires commonly used to assess quality of life among individuals with cancer. For example, the terms ‘EORTC QLQC’ and ‘European Organization’ refer to the European Organization for Research and Treatment of Cancer Quality of Life Questionnaire (EORTC QLQ-C30), which assesses physical functioning, role functioning, emotional, cognitive, and social functioning, as well as symptoms and global health status. The keyword ‘Functional assessment’ indicates the Functional Assessment of Cancer Therapy (FACT) questionnaire. The FACT-G (General) includes domains such as physical, social, emotional, and functional well-being, while the FACT-B (Breast cancer-specific) includes additional breast cancer-related questions related to body image and sexual function. Lastly, the term ‘Hospital anxiety depression’ refers to the Hospital Anxiety and Depression Scale (HADS), a widely used instrument for measuring psychological symptoms of anxiety and depression.

#### 3.1.2. Topic 2: Cancer Treatment and Health-Related Fitness

The keywords included in this topic reflect elements of research design, intervention type, target patient population, and measured outcomes. Terms such as ‘randomized trial, baseline postintervention, and aerobic resistance’ demonstrate that many studies utilized randomized controlled trial designs involving aerobic and resistance exercise interventions. The terms such as ‘adjuvant chemotherapy, adjuvant treatment, neoadjuvant chemotherapy, and chemotherapy treatment’ show that these interventions were conducted on individuals undergoing active treatment (e.g., chemotherapy). Moreover, this topic encompasses a variety of health-related fitness outcomes, including ‘walk test, body composition, physical performance, heart rate, functional capacity, aerobic capacity, minute walk, functional assessment, oxygen consumption, and peak oxygen’. Several other outcomes include ‘adverse event, patient-reported outcomes, and activity level’.

#### 3.1.3. Topic 3: Supportive Care and Psychosocial Factors (Qualitative)

The keywords in this topic reflect the common use of qualitative research designs and highlight the types of outcomes examined. Terms such as ‘semi-structured interview, thematic analysis, content analysis, and barrier facilitator’ indicate widely used qualitative data collection, outcome, and analysis methods. Supportive care-related keywords, such as ‘social support, supportive care, care need, and healthcare professional’, suggest that many studies focused on supportive care interventions or measured BCS’ supportive care needs. Consistent with the nature of qualitative research, these studies addressed psychological outcomes (e.g., psychological well-being, distress, and fear of recurrence), rather than physiological outcomes. Additionally, Tai Chi and dragon boat paddling were frequently used as intervention strategies among BCS. The topic also includes emerging studies focusing on young adult population and those conducted during the COVID-19 pandemic.

#### 3.1.4. Topic 4: Survivorship, Palliative Care, and Integrative Medicine

This topic includes keywords about survivorship care that utilizes integrative medicine. ‘Survivorship care, palliative care, supportive care, complementary alternative medicine, integrative oncology, integrative medicine’ indicate the inclusion of complementary and integrative care programs in the management of BCS. Additionally, terms such as ‘care provider, healthcare, primary care, and clinical practice’ indicate healthcare providers and clinical practice. Keywords, such as ‘risk factor, cardiovascular disease, chronic disease, cardiovascular risk, menopausal symptoms, and vasomotor symptoms’ points to a focus on common comorbidities, associated risk factors, and symptoms experienced by BCS. Furthermore, ‘survival rate’ and ‘United States’ are frequently used in background contexts, suggesting an emphasis on the rising survival rates of breast cancer and the predominance of U.S.-based statistics and evidence in the literature.

#### 3.1.5. Topic 5: Physical Activity and Sedentary Behaviors

This topic includes many keywords related to PA and sedentary behavior, such as ‘activity level, sedentary behavior, activity guideline, sedentary time, activity tracker, meet guideline, self-reported physical, daily step, regular physical, step count, and activity MVPA (moderate-to-vigorous PA)’. The term ‘activity tracker’ indicates a measuring tool and ‘daily step, step count, and MVPA’ indicate sub-categories of PA assessment. PA guidelines are commonly used to provide the cutoff for physical activity levels and assess adherence. Keywords such as ‘social cognitive’ and ‘cognitive theory’ reflect the application of health behavior change models and theories. The social cognitive theory includes sub-dimensions, such as self-efficacy, outcome expectations, observational learning/modeling, reciprocal determinism, self-regulation, reinforcement, and behavioral capability, and they are used as outcome variables in exercise intervention studies or as a tool for developing intervention programs to improve health behaviors. Additionally, ‘Posttraumatic growth’ appeared as an outcome variable in many studies, and ‘active treatment’ indicates that many studies have been conducted among BCS undergoing active treatment.

#### 3.1.6. Topic 6: Upper Limb-Related Side Effects

Most of the keywords in this topic pertained to lymphedema and its associated treatments. Terms such as ‘upper limb, upper extremity, cancer-related lymphedema, lymph node, arm volume, node dissection, limb volume, axillary lymph, disability arm, arm lymphedema, chronic pain, and volume difference’ are indicative of lymphedema. Breast cancer-related lymphedema is a common side effect of breast cancer treatment. It is a chronic, progressive condition characterized by the accumulation of protein-rich lymphatic fluid in the ipsilateral upper limb, breast, or chest wall, resulting from lymphatic system damage caused by cancer treatments (e.g., axillary lymph node dissection). ‘Complex decongestive’ refers to complex decongestive therapy (CDT), which is considered the gold-standard, multi-component conservative management strategy for lymphedema. Keywords such as ‘Manual lymphatic’ and ‘lymphatic drainage’ indicate manual lymphatic drainage, a core component of CDT. In addition, ‘upper limb, range motion, upper extremity, arm shoulder, shoulder range, disability arm, shoulder hand, and chronic pain’ are related to restricted shoulder and upper limb mobility, which is another common and clinically significant side effect of breast cancer treatment.

#### 3.1.7. Topic 7: Cancer-Related Fatigue and Symptoms

This topic includes keywords associated with common patient-reported symptoms experienced by BCS. Terms such as ‘Cancer-related fatigue, fatigue sleep, fatigue scale, fatigue depression, fatigue symptom, pain fatigue, fatigue inventory, fatigue treatment’ are related to fatigue, which is the most commonly reported symptom among BCS. ‘Sleep disturbance, sleep index, Pittsburgh sleep, cognitive function, cognitive impairment, cognitive functioning, depressive symptom, symptom burden, symptom cluster’ demonstrate other prevalent symptoms and associated assessment tools in BCS. Cognitive impairment, depressive symptoms, and sleep disturbance are frequently reported by BCS, and the Pittsburgh Sleep Quality Index (PSQI) is a questionnaire to assess sleep quality. The term ‘symptom cluster’ refers to two or more concurrent symptoms that are interrelated, occur together, and may have additive or synergistic effects on patient outcomes. ‘Functional assessment’ refers to the FACT questionnaire explained in Topic 1. ‘Effect yoga’ suggest that yoga has been used as an intervention to alleviate cancer-related fatigue, improve sleep quality, and enhance overall HRQoL.

#### 3.1.8. Topic 8: Epidemiological and Clinical Factors

This topic encompasses a mixture of different themes, with a primary focus on epidemiological and clinical factors based on keywords such as ‘risk factor, demographic clinical, body mass, health behavior, young age, activity level, since diagnosis, and time diagnosis’. ‘Body mass’ indicates body mass index (BMI), a well-established risk factor for breast cancer and a frequently used covariate in cancer studies. ‘Young age’ refers to younger age at breast cancer diagnosis in most contexts. Terms such as ‘since diagnosis’ and ‘time diagnosis’ refer to time since diagnosis which is another common covariate. ‘Social support’, which also appeared in other topics, indicates that psychosocial factors are highlighted as key in managing psychological outcomes as well as PA. Terms such as ‘psychological distress, health status, and depressive symptom’ are commonly used outcomes that are examined alongside quality of life. ‘Psychological distress’ is often cited as a primary symptom experienced by cancer patients following diagnosis. ‘Health status’ typically refers to global health status, one of the core components of the EORTC QLQ-C30. Keywords such as ‘newly diagnose’ and ‘first year’ indicate that many studies have focused on BCS in the early period following diagnosis. ‘Newly diagnose’ refers to newly diagnosed patients, and ‘first year’ refers to first year post-diagnosis or post-treatment. Lastly, several studies included in this topic compare BCS to the ‘general population’.

#### 3.1.9. Topic 9: Side Effects of Cancer Treatment

This topic includes keywords related to adjuvant therapies and their associated adverse events. For example, terms such as ‘adjuvant treatment’ and ‘aromatase inhibitor’ indicate the use of hormone therapy involving aromatase inhibitors. Keywords such as ‘adverse effects, adverse event, skeletal muscle, muscle mass, bone loss, bone mineral, bone health, mineral density, and joint pain’ are related to musculoskeletal events, which are common side effects of aromatase inhibitor therapy. In addition, ‘peripheral neuropathy’ and ‘chemotherapy induced peripheral’ indicate chemotherapy-induced peripheral neuropathy, another common adverse effect experienced by BCS undergoing treatment. ‘Bone health’ and ‘joint pain’ also relate to ‘bone metastasis’. Keywords such as ‘clinical practice’ and ‘practice guideline’ demonstrate that many studies have aimed to collect evidence and develop clinical practice guidelines for managing treatment-related adverse events. Emerging areas of interest in this topic include ‘oxidative stress’ and ‘gut microbiota’, which have been linked to tumor progression, treatment response, HRQOL, and exercise interventions in the context of breast cancer care.

#### 3.1.10. Topic 10: Weight Management

Keywords in this topic are associated with weight management, particularly body composition and dietary behaviors, both of which are established risk factors for breast cancer recurrence. ‘Weight loss, body composition, body mass, body weight, weight gain, overweight obese, body fat, waist circumference, and change body’ relate to various aspects of body composition. Meanwhile, ‘healthy lifestyle, lifestyle behavior, lifestyle change, diet physical, fruit vegetable, healthy diet, dietary intake, and health behavior’ reflect dietary and lifestyle behaviors beyond PA. Additionally, several studies focused on ‘African American’ women, a population with a relatively higher prevalence of obesity and sub-optimal dietary patterns, underscoring the need for targeted interventions in this group.

### 3.2. Research Trends

**Figure 2:**
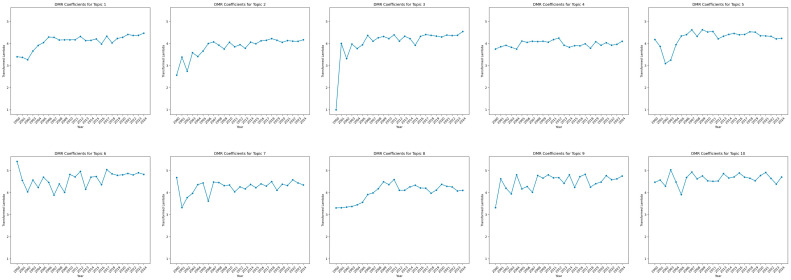
Research Trends. Note: Panels are arranged left-to-right, top-to-bottom: Topic 1 (**upper-left**) through Topic 5 (**upper-right**) and Topic 6 (**lower-left**) through Topic 10 (**lower-right**); higher values reflect a stronger association between a given topic and the corresponding year, indicating increased research interest in that topic over time; the figure shows upward trends (Topics 1, 2, 3, 8, and 9) and stable trends (Topics 4, 5, 6, 7, and 10).

Figure 2 depicts the topic trends derived from DMR analysis (2000–2024), where higher values indicate stronger associations for each topic in a given year. Table 2 shows the average slope for each topic. By comparing recent years to the baseline (Year 2000) and analyzing emerging patterns, we observed diverse temporal patterns across the ten topics. Notably, none of the average slopes were negative, suggesting that research activity in this field has remained steady or even increased over time. Topics 1, 2, 3, 8, and 9 presented upper trends, whereas Topics, 4, 5, 6, 7, and 10 remained comparatively stable, without a consistent directional trend.

Topic 1 (Quality of Life and Well-being) showed a notable upward trajectory, maintaining consistently high representation in recent years. Topic 2 (Cancer Treatment and Health-Related Fitness) exhibited a steady increase over time, reflecting sustained interest in physical fitness interventions during cancer treatment. Topic 3 (Supportive Care and Psychosocial Factors) experienced a sharp rise in the early 2000s and remained relatively high, suggesting a long-standing research focus on psychosocial outcomes and qualitative methodologies. Topic 8 (Epidemiological and Clinical Factors) displayed a steady overall increase, suggesting sustained research priorities concerning clinical and demographic variables. Topic 9 (Side Effects of Cancer Treatment) fluctuated over time but exhibited an overall upward trend.

Meanwhile, Topic 4 (Survivorship, Palliative Care, and Integrative Medicine) remained relatively stable, with slight increases in recent years, indicating growing attention to integrative care within survivorship contexts. Topic 5 (Physical Inactivity and Sedentary Behaviors) increased notably at first and then plateaued, suggesting that sedentary behavior has remained a consistent area of interest. In contrast, Topic 6 (Upper Limb-Related Side Effects) demonstrated more variability but stabilized recently, potentially indicating reduced emphasis or topic saturation. Topic 7 (Cancer-Related Fatigue and Symptoms) rose initially before stabilizing, signifying continued interest in symptom management. Lastly, Topic 10 (Weight Management) showed moderate variation, but a steady trend overall, suggesting continuous focus on obesity and lifestyle interventions in breast cancer survivorship research.

## 4. Discussion

Breast cancer is a significant global health issue for women, and HRQoL has consistently received attention across all stages of breast cancer trajectory. Regular participation in PA has been widely recognized as an effective strategy for improving HRQoL in this population. The present study applied a DMR-based text-mining approach to explore key research topics and their temporal trends in research pertaining to HRQoL and PA among BCS. Through identifying 10 distinct topics, our findings highlighted the thematic diversity of the literature, encompassing a wide range of study designs, including RCTs, qualitative studies, and epidemiological research, as well as a broad spectrum of health-related outcomes. These findings reflect the multi-faceted nature of research on PA and HRQoL among BCS, highlighting the integration of diverse clinical and behavioral approaches.

Among the topics that demonstrated increasing trends, Cancer Treatment and Health-Related Fitness (Topic 2) showed the growing number of RCTs that have examined the effects of exercise interventions during active cancer treatment. Most early exercise oncology research focused on post-treatment survivorship [9], driven by the prevailing belief that patients undergoing chemotherapy or other systemic therapies would not be able to tolerate exercise [10]. Recent literature, however, increasingly challenges this notion. Accumulating evidence shows that structured aerobic and resistance training during treatment is not only feasible and safe but also provides multiple benefits, including improvements in fitness, physical function, and psychological well-being [11,12,13]. Moreover, PA may enhance treatment tolerance and completion rates by mitigating exhaustion and toxicities that often lead to dose reductions or treatment delays [14]. The findings suggest a paradigm shift, positioning exercise as an integrative component of oncology care rather than a supportive or additional component in post-treatment periods.

Side Effects of Cancer Treatment (Topic 9) demonstrated increased scholarly attention to the role of exercise in managing specific adverse effects, particularly, musculoskeletal symptoms induced by endocrine therapy and chemotherapy-induced peripheral neuropathy (CIPN). Exercise interventions have been shown to significantly reduce joint pain and stiffness in patients on aromatase inhibitors [15], a major cause of non-adherence to hormone therapy. Similarly, preliminary trials and meta-analyses suggest that exercise might alleviate CIPN symptoms [16,17], through mechanisms involving improved circulation, neuromuscular function, and anti-inflammatory effects. Together these two emerging topics (Topics 2 and 9) underscore the growing recognition of exercise as a non-pharmacologic therapeutic modality capable of mitigating treatment-related side effects, maintaining functional capacity, and enhancing the tolerability and delivery of cancer therapies—not merely as a means to improve fitness or quality of life.

Epidemiological and Clinical Factors (Topic 8) included a diverse array of keywords. Epidemiological research was one of the sub-topics. In addition to RCTs, a substantial body of epidemiological research has contributed to identifying patterns, risk factors, and vulnerable populations across the cancer care continuum [5,18,19,20,21]. This highlights the methodological breadth of the field and underscores the value of large-scale, population-based data in complementing findings from intervention-based research. Another keyword was newly diagnosed BCS, particularly those in the period between diagnosis and the initiation of major treatment (e.g., surgery or chemotherapy). Similarly to patients undergoing treatment, this group has been largely overlooked in cancer research, which has traditionally focused on cancer survivors’ post-treatment. However, recent evidence suggests that this early ‘pre-treatment’ window is clinically meaningful [22]. Early interventions such as PA or psychosocial support during this period may improve physical function, reduce treatment-related complications, enhance treatment readiness, and potentially influence cancer prognosis [23,24,25]. Courneya’s group recently published two papers from the Alberta moving beyond breast cancer (AMBER) cohort study [26,27], demonstrating that higher levels of physical fitness were significantly associated with better quality of life and fewer cancer-related symptoms in newly diagnosed BCS.

Another notable keyword in Topic 8 was ‘young age’. While the overall incidence of breast cancer increases with age, accumulating epidemiological studies have reported a gradual shift toward earlier age at diagnosis [28,29,30], particularly among women in their 30s and early 40s. This trend is especially concerning, as younger patients are more likely to have more aggressive tumor sub-types, poorer prognoses, and more complex psychosocial challenges [28,31,32]. Anders et al. (2009) found that being younger than 35 years is an independent predictor of both recurrence and overall mortality, underscoring the need for tailored survivorship care plans for younger BCS [28]. As such, an increasing number of studies have begun to focus on young BCS and explore tailored intervention programs for them. However, more research is still warranted, as the majority of previous studies have primarily focused on older adults [33]. Given the increasing prevalence of breast cancer among young adults, it is essential to address their unique challenges and concerns, such as fertility preservation, relationship status, career disruption, and long-term body image, which are distinct from those of older adults and significantly influence overall QoL [28].

In addition to the increasing number of quantitative studies, Supportive Care and Psychosocial Factors (Topic 3) highlights a growing body of qualitative research that explores the lived experiences of individuals living with and beyond breast cancer in terms of PA and quality of life. Unlike quantitative methods, which are often limited to predefined variables and outcomes, qualitative approaches allow for a deeper exploration of BCS’ subjective experiences, including fear of recurrence and perceived barriers and facilitators to engaging in PA. For instance, a focus group study by Sander et al. (2012) presented that fears of triggering or worsening lymphedema, along with uncertainty about safe and effective exercise, were major factors influencing activity choices among BCS, highlighting the need for individualized, evidence-based exercise prescriptions [34]. Understanding the thoughts, emotions, and lived experiences of breast cancer patients is critical for designing and implementing patient-oriented interventions that meet the needs of both researchers and participants [35,36]. To achieve this, interpersonal relationships, communication with healthcare providers, and access to psychosocial resources would play an important role. Topic 3 demonstrated that the contribution of qualitative research has grown gradually, offering critical insights into aspects of the breast cancer experience that are often overlooked or insufficiently captured by quantitative approaches. Moreover, the growing emphasis on qualitative methodologies represents an important shift toward more holistic and person-centered research in the field of exercise oncology.

While several topics exhibited an increasing trend, the others remained stable without topics with declining trend. Specifically, Survivorship, Palliative care, and Integrative Medicine (Topic 4) demonstrated the role of PA as a core component within supportive care and integrative medicine programs for breast cancer patients. In this context, PA is embedded alongside other complementary approaches such as nutrition counseling, psychosocial support, stress management, and mind–body therapies [37,38]. This reflects a recognition of the multi-dimensional needs of BCS, which extend beyond disease eradication to include symptom management, functional restoration, and overall QoL. Moreover, the term ‘United States’ in this topic suggests that the majority of studies were conducted in United States cohorts. Evidence shows that U.S. trials are dominated by highly educated, non-Hispanic White survivors with good access to care, while racial/ethnic minorities and rural cancer survivors remain under-represented [39,40]. Therefore, there is more need for research involving diverse racial and ethnic populations as well as rural BCS to enhance generalizability and address health disparities.

Both Topics 6 (Upper Limb-Related Side Effects) and 7 (Cancer-Related Fatigue and Symptoms) represent common and burdensome symptoms experienced by BCS, such as upper limb-related side effects such as lymphedema (Topic 6) and cancer-related fatigue, sleep disturbances, and cognitive complaints (Topic 7). These symptoms are well known to negatively affect HRQoL [41,42], but they can also be mitigated through exercise or PA [42,43]. These findings suggest that exercise plays a critical role not only in physical rehabilitation but also in symptom management, highlighting its relevance across multiple domains of survivorship care.

Lastly, Weight Management (Topic 10) focused on healthy weight regulation, including keywords related to body composition and dietary behaviors. Given the well-established association between overweight/obesity and poorer breast cancer prognosis and survival [44,45], this remains a critical and consistently studied area. Increasing PA is one of the most effective strategies for preventing weight gain [46,47], making it a key component of long-term weight management interventions. As such, this topic reinforces the importance of integrating lifestyle management, including multi-component programs that combine PA and diet into routine survivorship care.

This DMR-based text-mining study provides valuable, data-driven insights into how PA and HRQoL research among BCS has evolved since 2000. By analyzing the entire corpus of abstracts and incorporating document-level metadata (i.e., publication year), our study objectively uncovered latent topics across the literature [7,8], minimizing the selection bias typical of manual reviews. The longitudinal nature of DMR enables us to plot topic prevalence by year, facilitating the visualization of trends related to interest in QoL while revealing emergent or previously overlooked areas and pinpointing under-explored research questions for future investigations. This study’s findings offer practical implications for both researchers and healthcare providers, helping them identify new directions and bridge research gaps to enhance BCS outcomes. In particular, strengthening collaborations between research teams and healthcare providers can improve access to BCS and deepen the understanding of survivors’ lived experiences. These thematic insights can also help develop a conceptual framework for communicating complex survivorship issues and inform evidence-based decision making in healthcare and policy, leading to more effective strategies that address survivorship needs in a timely manner.

The findings offer actionable guidance for survivorship care planning. First, the growing evidence that combined aerobic and resistance training is safe and feasible during active treatment may encourage oncology teams to consider integrating personalized exercise prescriptions into routine chemotherapy and radiotherapy pathways, helping to preserve cardiorespiratory fitness, muscle strength, and psychological well-being. Second, increasing attention to endocrine therapy joint pain and CIPN highlights exercise as a promising non-pharmacologic option for symptom relief; clinicians may consider low impact strength and balance routines, adjusting intensity based on patient-reported outcomes. Third, emerging research on the pre-treatment window suggests that even brief programs of moderate aerobic activity and functional strength exercise may improve surgical readiness and early HRQoL, warranting consideration soon after diagnosis. Finally, the continued emphasis on upper-limb symptoms, cancer-related fatigue, cognitive complaints, and weight management underscores the need to embed exercise and broader lifestyle goals into survivorship care plans—particularly multi-component programs that couple PA with dietary counseling. This holistic approach not only strengthens our ability to align evolving breast cancer research with real-world priorities of survivors, clinicians, and policymakers, but also fosters a more unified dialog among professionals committed to advancing breast cancer survivorship care.

Despite these numerous strengths and contributions to breast cancer survivorship research, the findings of this study must be interpreted cautiously for several reasons. First, because DMR remains an unsupervised technique for topic generation, researchers must manually assign labels to the topic clusters—a process that introduces subjectivity and depends on the interpretive skill of domain experts. However, we mitigated this limitation by implementing a systematic labeling procedure, which included an iterative review, consensus-building among multiple authors, and cross-referencing with established survivorship terminology. Second, although DMR leverages metadata, some topics may appear too broad or too narrow, particularly if top-ranked keywords are highly generic. For example, Topics 1 (Quality of Life and Well-being) and 5 (Physical Activity and Sedentary Behavior) may have been extracted because their keywords lists are dominated by our search terms—generic HRQoL measures (Topic 1) and PA terminology (Topic 5), yielding limited thematic specificity. Future research could reduce this redundancy by applying hierarchical topic modeling approaches—such as Hierarchical Latent Dirichlet Allocation—which capture both broad parent themes and their sub-topics. This approach enables more nuanced distinctions between closely related constructs and brings associated sub-terms into sharper focus. Finally, topic models merely identify language patterns in the literature and cannot assess methodological quality or imply any causal relationships in survivorship research. The presence of a term indicates thematic prevalence, not necessarily the rigor or impact of the underlying studies.

Surprisingly, despite the growing interest in digital health [48,49,50], keywords related to remote interventions, mobile applications, mobile health, or web-based exercise programs were not prominently identified in this analysis. As our search strategy focused broadly on BCS, PA, and HRQoL. The absence of these terms likely reflects two factors: (1) the relatively recent emergence of these approaches and their limited representation in the literature to date, and (2) the wide range of terms used to refer to technology-based interventions, which may reduce the apparent frequency of any single term in large scale text analyses. Given their practical utility, scalability, and alignment with current technological trends, future research should prioritize the development and evaluation of digital and remotely delivered exercise interventions tailored for breast cancer populations [51]. Researchers aiming to map this rapidly evolving area with DMR or related topic modeling methods should develop search strategies that deliberately incorporate a wide range of technology-related terms (e.g., digital health, eHealth, mHealth, telehealth, app-based, web-based) and account for terminology variations across disciplines and countries. This approach ensures a more comprehensive capture of relevant literature. Moreover, future research should move beyond the general knowledge that PA is beneficial for cancer patients and instead focus on identifying optimal and tailored exercise prescriptions, including type, intensity, volume, and frequency, based on clinical characteristics, treatment phase, and individual needs. Such precision-based approaches will enhance the clinical applicability of exercise oncology. Finally, the current literature remains predominantly centered on White, non-Hispanic populations [52,53], underscoring the need for more inclusive research that considers racial and ethnic diversity, including underserved populations (e.g., rural, minority). Future studies should prioritize effective recruitment strategies that engage under-represented populations through community partnerships and culturally tailored approaches. Moreover, employing mixed methods or community-based participatory research approaches may enhance the relevance and acceptability of interventions in diverse populations. Understanding how sociocultural, genetic, and systemic factors influence PA behaviors and treatment responses among diverse BCS is critical for addressing health disparities in cancer care.

## 5. Conclusions

This study is the first to apply advanced text-mining techniques (i.e., DMR) to uncover and track hidden themes in breast cancer survivorship literature on PA/exercise and HRQoL. The results demonstrated that exercise oncology research has diversified well beyond post-treatment survivorship to encompass the full cancer trajectory and addresses a wide range of clinical and psychosocial outcomes. The resulting themes offer a roadmap for further scientific inquiry and highlight practical avenues. The 10 data-driven topics identified in this study outline current research topics and clear directions for future investigation and practice on PA and HRQoL among BCS. To ensure interventions are equitable and broadly acceptable, future research should prioritize the inclusion of BCS from racially, socioeconomically, and geographically diverse backgrounds—groups that remain under-represented in the current literature. By pinpointing emerging priorities and evidence gaps, the findings give researchers, clinicians, and policymakers a strategic guide for optimizing PA-based supportive care and, ultimately, improving the quality of life for women living with breast cancer.

## Figures and Tables

**Table 1:** Research Topics.

Topic 1	Topic 2	Topic 3	Topic 4	Topic 5
Quality of Life and Well-Being	Cancer Treatment and Health-Related Fitness	Supportive Care and Psychosocial Factors (Qualitative)	Survivorship, Palliative Care and Integrative Medicine	Physical Activity and Sedentary Behaviors
body_image	adverse_event	social_support	risk_factor	activity_level
eortc_qlqc	adjuvant_chemotherapy	supportive_care	health_care	sedentary_behavior
functional_assessment	aerobic_resistance	semistructured_interview	survivorship_care	activity_guideline
european_organization	walk_test	care_need	effect_treatment	social_cognitive
physical_psychological	body_composition	tai_chi	cardiovascular_disease	sedentary_time
physical_well-being	physical_performance	physical_psychological	menopausal_symptom	activity_tracker
global_health	heart_rate	young_adult	diagnosis_treatment	active_treatment
health_status	functional_capacity	covid_pandemic	palliative_care	meet_guideline
social_functioning	patientreported_outcome	dragon_boat	chronic_disease	posttraumatic_growth
hospital_anxiety_depression	aerobic_capacity	thematic_analysis	supportive_care	activity_treatment
**Topic 6**	**Topic 7**	**Topic 8**	**Topic 9**	**Topic 10**
**Upper Limb-Related Side Effects**	**Cancer-Related Fatigue and Symptoms**	**Epidemiological and Clinical Factors**	**Side Effects of Cancer Treatment (Neuropathy/Bone Health)**	**Weight Management**
upper_limb	cancerrelated_fatigue	risk_factor	aromatase_inhibitor	weight_loss
cancerrelated_lymphedema	sleep_disturbance	social_support	skeletal_muscle	body_composition
range_motion	cognitive_function	body_mass	systematic_review	body_mass
lymph_node	cognitive_impairment	health_behavior	peripheral_neuropathy	body_weight
upper_extremity	depressive_symptom	year_diagnosis	adverse_effect	weight_gain
arm_volume	fatigue_sleep	old_adult	bone_loss	african_american
lymphatic_drainage	symptom_burden	activity_level	joint_pain	healthy_lifestyle
node_dissection	fatigue_scale	psychological_distress	chemotherapyinduced_peripheral	overweight_obese
arm_shoulder	fatigue_depression	since_diagnosis	clinical_practice	lifestyle_behavior
shoulder_range	fatigue_symptom	newly_diagnose	practice_guideline	weight_management

Note: The topic order is arbitrarily discovered by the DMR model; eortc_qlqc refers to the EORTC QLQ-C30, a cancer-specific quality of life questionnaire.

**Table 2:** Average yearly change by topic.

Topic	Slope Coefficient
1	0.034
2	0.044
3	0.052
4	0.003
5	0.023
6	0.017
7	0.016
8	0.036
9	0.023
10	0.008

## Data Availability

The dataset analyzed during the current study is available from the first author on reasonable request.

## Supplementary Materials

The following supporting information can be downloaded at: https://www.mdpi.com/article/10.3390/jcm14165615/s1, Table S1: PubMed search queries (2 July 2025), Table S2: Research topics (Full Keywords), Table S3: Example articles for each identified topic.

## Abbreviations

PA = physical activity, HRQoL = health-related quality of life, BCS = breast cancer survivors, DMR = Dirichlet-Multinomial Regression, RCT = randomized controlled trial, EORTC QLQ-C30 = European Organization for Research and Treatment of Cancer Quality of Life Questionnaire, FACT = Functional Assessment of Cancer Therapy, FACT-G = Functional Assessment of Cancer Therapy-General, FACT-B = Functional Assessment of Cancer Therapy-Breast, HADS = Hospital Anxiety and Depression Scale, CDT = Complex Decongestive Therapy, MVPA = moderate-to-vigorous physical activity, PSQI = Pittsburgh Sleep Quality Index, BMI = body mass index, CIPN = chemotherapy-induced peripheral neuropathy, AMBER = Alberta Moving Beyond Breast Cancer, eHealth = electronic health, mHealth = mobile health.
