# Supplementary material for: Landscape of Physical Activity and Quality of Life Research in Breast Cancer Survivors: Topic Modeling Analysis
Source: J Clin Med. 2025 Aug 8;14(16):5615. doi: 10.3390/jcm14165615 (PMC12386802; doi:10.3390/jcm14165615)
Supplement: Supplementary file 1 [file jcm-14-05615-s001.zip › jcm-3753255-Supplementary.pdf]

Supplementary Table S1. PubMed Search queries (2/7/2025)

	Main Themes	Search Terms	N
1	Breast cancer survivors	(“breast cancer”[tiab] OR “breast neoplasm*”[tiab] OR “breast carcinoma*”[tiab] OR “breast tumo*”[tiab])	403,319
2	Physical activity/ exercise	(((“physical activit*”[tiab] OR exercise*[tiab] OR fitness[tiab]) AND (program*[tiab] OR intervention*[tiab] OR training*[tiab] OR therap*[tiab])) OR (“physical exercise*”[tiab] OR “physical training*”[tiab] OR “physical activity behavio*”[tiab] OR “exercise behavio*”[tiab]))	268,753
3	Quality of life	(“quality of life” [tiab] OR “health-related quality of life”[tiab] OR “life quality” [tiab] OR “well-being” [tiab])	547,618
4		#1 AND #2 AND #3	1,403
5		(#1 AND #2 AND #3) AND (English[la]) AND (2000:2024[dp]) AND (“Journal Article”[pt])	1,344

Note: N: numbers; For keywords representing breast cancer, we did not include the term “survivor,” in alignment with the current recommended definitions of the NIH National Cancer Institute.

Table S2. Research Topics (Full Keywords)

Topic 1	Topic 2	Topic 3	Topic 4	Topic 5
**Quality of Life & Well-being**	**Cancer Treatment & Health-related Fitness**	**Supportive Care & Psychosocial Factors (Qualitative)**	**Survivorship, Palliative care & Integrative Medicine**	**Physical Activity & Sedentary Behaviors**
body_image<br>eortc_qlqc<br>functional_assessment<br>european_organization<br>physical_psychological<br>physical_wellbeing<br>global_health<br>health_status<br>social_functioning<br>hospital_anxiety_depression<br>short_form<br>return_work<br>anxiety_depression_scale<br>treatment_questionnaire<br>emotional_wellbeing<br>sexual_function<br>functional_wellbeing<br>physical_emotional<br>emotional_functioning<br>health_survey	adverse_event<br>adjuvant_chemotherapy<br>aerobic_resistance<br>walk_test<br>body_composition<br>physical_performance<br>heart_rate<br>functional_capacity<br>patientreported_outcome<br>aerobic_capacity<br>minute_walk<br>activity_level<br>functional_assessment<br>adjuvant_treatment<br>randomize_trial<br>neoadjuvant_chemotherapy<br>oxygen_consumption<br>baseline_postintervention<br>chemotherapy_treatment<br>peak_oxygen	social_support<br>supportive_care<br>semistructured_interview<br>care_need<br>tai_chi<br>physical_psychological<br>young_adult<br>covid_pandemic<br>dragon_boat<br>thematic_analysis<br>participant_report<br>psychological_wellbeing<br>psychological_distress<br>content_analysis<br>fear_recurrence<br>barrier_facilitator<br>healthcare_professional<br>physical_emotional<br>physical_psychosocial<br>live_beyond	risk_factor<br>health_care<br>survivorship_care<br>effect_treatment<br>cardiovascular_disease<br>menopausal_symptom<br>diagnosis_treatment<br>palliative_care<br>chronic_disease<br>supportive_care<br>united_state<br>complementary_alternative_medicine<br>clinical_practice<br>care_provider<br>primary_care<br>vasomotor_symptom<br>integrative_oncology<br>cardiovascular_risk<br>integrative_medicine<br>survival_rate	activity_level<br>sedentary_behavior<br>activity_guideline<br>social_cognitive<br>sedentary_time<br>activity_tracker<br>active_treatment<br>meet_guideline<br>posttraumatic_growth<br>activity_treatment<br>health_outcome<br>change_physical<br>selfreported_physical<br>daily_step<br>measure_physical<br>regular_physical<br>demographic_medical<br>step_count<br>activity_mvpa<br>cognitive_theory

Topic 6	Topic 7	Topic 8	Topic 9	Topic 10
**Upper Limb-related Side Effects**	**Cancer-related Fatigue and Symptoms**	**Epidemiological and Clinical Factors**	**Side Effects of Cancer Treatment (Neuropathy/ Bone Health)**	**Weight Management**
upper_limb<br>cancerrelated_lymphedema<br>range_motion<br>lymph_node<br>upper_extremity<br>arm_volume<br>lymphatic_drainage<br>node_dissection<br>arm_shoulder<br>shoulder_range<br>limb_volume<br>complex_decongestive<br>manual_lymphatic<br>axillary_lymph<br>disability_arm<br>shoulder_hand<br>arm_lymphedema<br>month_surgery<br>chronic_pain	cancerrelated_fatigue<br>sleep_disturbance<br>cognitive_function<br>cognitive_impairment<br>depressive_symptom<br>fatigue_sleep<br>symptom_burden<br>fatigue_scale<br>fatigue_depression<br>fatigue_symptom<br>pain_fatigue<br>fatigue_inventory<br>cognitive_functioning<br>head_neck<br>functional_assessment<br>fatigue_treatment<br>sleep_index<br>pittsburgh_sleep<br>effect_yoga<br>symptom_cluster	risk_factor<br>social_support<br>body_mass<br>health_behavior<br>year_diagnosis<br>old_adult<br>activity_level<br>psychological_distress<br>since_diagnosis<br>newly_diagnose<br>health_status<br>general_population<br>young_age<br>year_postdiagnosis<br>demographic_clinical<br>month_postdiagnosis<br>old_age<br>depressive_symptom<br>first_year<br>time_diagnosis	aromatase_inhibitor<br>skeletal_muscle<br>systematic_review<br>peripheral_neuropathy<br>adverse_effect<br>bone_loss<br>joint_pain<br>chemotherapyinduced_peripheral<br>clinical_practice<br>practice_guideline<br>adjuvant_treatment<br>adverse_event<br>bone_mineral<br>risk_factor<br>bone_health<br>mineral_density<br>bone_metastasis<br>muscle_mass<br>oxidative_stress<br>gut_microbiota	weight_loss<br>body_composition<br>body_mass<br>body_weight<br>weight_gain<br>african_american<br>healthy_lifestyle<br>overweight_obese<br>lifestyle_behavior<br>weight_management<br>body_fat<br>lifestyle_change<br>diet_physical<br>fruit_vegetable<br>waist_circumference<br>risk_recurrence<br>healthy_diet<br>dietary_intake<br>health_behavior<br>change_body

volume_difference				

Note: The topic order is arbitrary discovered by the DMR model; eortc_qlqc refers to the EORTC QLQ-C30, a cancer-specific quality of life questionnaire.

Table S3. Example Articles for Each Identified Topic

Topic	Example Article
1	Sodergren SC, Copson E, White A, Efficace F, Sprangers M, Fitzsimmons D, Bottomley A, Johnson CD. Systematic review of the side effects associated with anti-HER2-targeted therapies used in the treatment of breast cancer, on behalf of the EORTC quality of life group. Targeted oncology. 2016 Jun;11(3):277-92.
2	Williams AF, Vadgama A, Franks PJ, Mortimer PS. A randomized controlled crossover study of manual lymphatic drainage therapy in women with breast cancer-related lymphoedema. European journal of cancer care. 2002 Dec;11(4):254-61.
3	Manuel JC, Burwell SR, Crawford SL, Lawrence RH, Farmer DF, Hege A, Phillips K, Avis NE. Younger women's perceptions of coping with breast cancer. Cancer nursing. 2007 Mar 1;30(2):85-94.
4	Gulde I, Oldervoll LM, Martin C. Palliative cancer patients’ experience of physical activity. Journal of palliative care. 2011 Dec;27(4):296-302.
5	Delrieu L, Pérol O, Fervers B, Friedenreich C, Vallance J, Febvey-Combes O, Pérol D, Roitmann E, Dufresne A, Bachelot T, Heudel PE. A personalized physical activity program with activity trackers and a mobile phone app for patients with metastatic breast cancer: protocol for a single-arm feasibility trial. JMIR Research Protocols. 2018 Aug 30;7(8):e10487.
6	Sagen A, Kaaresen R, Sandvik L, Thune I, Risberg MA. Upper limb physical function and adverse effects after breast cancer surgery: a prospective 2.5-year follow-up study and preoperative measures. Archives of physical medicine and rehabilitation. 2014 May 1;95(5):875-81.
7	Cai B, Allexandre D, Rajagopalan V, Jiang Z, Siemionow V, Ranganathan VK, Davis MP, Walsh D, Dai K, Yue GH. Evidence of significant central fatigue in patients with cancer-related fatigue during repetitive elbow flexions till perceived exhaustion. PloS one. 2014 Dec 22;9(12):e115370.
8	Leach CR, Weaver KE, Aziz NM, Alfano CM, Bellizzi KM, Kent EE, Forsythe LP, Rowland JH. The complex health profile of long-term cancer survivors: prevalence and predictors of comorbid conditions. Journal of cancer survivorship. 2015 Jun;9(2):239-51.
9	Niravath P. Aromatase inhibitor-associated arthralgia: how big is the problem and what can be done?. Current Breast Cancer Reports. 2016 Sep;8(3):135-8.
10	Gnagnarella P, Dragà D, Baggi F, Simoncini MC, Sabbatini A, Mazzocco K, Bassi FD, Pravettoni G, Maisonneuve P. Promoting weight loss through diet and exercise in overweight or obese breast cancer survivors (InForma): study protocol for a randomized controlled trial. Trials. 2016 Jul 28;17(1):363.
